# Evidence for UV-green dichromacy in the basal hymenopteran *Sirex noctilio* (Siricidae)

**DOI:** 10.1038/s41598-021-95107-2

**Published:** 2021-08-02

**Authors:** Quentin Guignard, Johannes Spaethe, Bernard Slippers, Martin Strube-Bloss, Jeremy D. Allison

**Affiliations:** 1 Department of Zoology and Entomology, Forestry and Agricultural Biotechnology Institute (FABI), University of Pretoria, Pretoria, 0002 South Africa; 2 Department of Behavioral Physiology and Sociobiology, Biozentrum, University of Würzburg, Am Hubland, Würzburg, Germany; 3 Department of Biochemistry, Genetics and Microbiology, Forestry and Agricultural Biotechnology Institute (FABI), University of Pretoria, Pretoria, 0002 South Africa; 4 Natural Resources Canada, Canadian Forest Service, Great Lakes Forestry Centre, 1219 Queen Street E, Sault Ste. Marie, ON P6A 2E5 Canada; 5 Present Address: Department of Biological Cybernetics, Faculty of Biology, Bielefeld University, 33615 Bielefeld, Germany

**Keywords:** Entomology, Molecular evolution, Colour vision

## Abstract

A precondition for colour vision is the presence of at least two spectral types of photoreceptors in the eye. The order Hymenoptera is traditionally divided into the Apocrita (ants, bees, wasps) and the Symphyta (sawflies, woodwasps, horntails). Most apocritan species possess three different photoreceptor types. In contrast, physiological studies in the Symphyta have reported one to four photoreceptor types. To better understand the evolution of photoreceptor diversity in the Hymenoptera, we studied the Symphyta *Sirex noctilio*, which belongs to the superfamily Siricoidea, a closely related group of the Apocrita suborder. Our aim was to (i) identify the photoreceptor types of the compound eye by electroretinography (ERG), (ii) characterise the visual opsin genes of *S. noctilio* by genomic comparisons and phylogenetic analyses and (iii) analyse opsin mRNA expression. ERG measurements revealed two photoreceptor types in the compound eye, maximally sensitive to 527 and 364 nm. In addition, we identified three opsins in the genome, homologous to the hymenopteran green or long-wavelength sensitive (LW) LW1, LW2 and ultra-violet sensitive (UV) opsin genes. The *LW1* and *UV* opsins were found to be expressed in the compound eyes, and *LW2* and *UV* opsins in the ocelli. The lack of a blue or short-wavelength sensitive (SW) homologous opsin gene and a corresponding receptor suggests that *S. noctilio* is a UV-green dichromate.

## Introduction

The ability to see colours, i.e. to discriminate between different wavelengths of light independent of intensity, provides insects with valuable information about their environment. Visual information, including shape, movement and colour of visual stimuli can induce adaptive behavioural responses. Dragonflies possess reduced antenna and auditory organs but well developed and enlarged compound eyes to visually locate their prey and mate^1^. Visual stimuli can also be important for mate recognition^2^, mate choice^3,4^, location of food^5,6^ or avoidance behaviour^3^. Insect colour vision may also play a role in more complex tasks such as counting^7^ and social learning^8^. Colour vision requires the presence of at least two different photoreceptor types tuned to different wavelengths of the light spectrum^9,10^. Sensitivity to different wavelengths can be under selective pressure and consequently varies among insects with different visual ecology^11–13^.

The spectral sensitivity of each photoreceptor is mainly determined by its light-sensitive visual pigment. Other factors can also influence the spectral sensitivity of the photoreceptor (e.g. coloured screening pigments), but none have been described for Hymenoptera^14–17^. The visual pigment consists of the opsin protein and a vitamin A-based chromophore^18^. The sensitivity of the photoreceptor to different wavelengths can be tuned by the opsin protein, which is called spectral tuning. There are three major groups of visual opsin genes in insects, named for the region of the spectrum they absorb; the ultraviolet (UV), blue or short wavelength (SW) and green or long wavelength (LW) sensitive opsins^9,16,19,20^. The LW group in Hymenoptera can be subdivided into the LW1 and the LW2 opsins, expressed in the compound eyes and the ocelli, respectively^21,22^.

The basis of colour vision appears to differ between Apocrita (ants, bees, wasps) and Symphyta (sawflies, woodwasps, horntails). All Apocrita investigated to date, except for the Ichneumonidae, possess one copy of the LW1, SW and UV visual opsin gene expressed in the green, blue and UV-sensitive photoreceptors, respectively^16,23,24^. In Symphyta, between one and four photoreceptor types have been reported, but no visual opsin gene has been characterised^23^. For example, evidence for an additional (fourth) red photoreceptor was found in *Tenthredo campestris*, *T. scrophulariae* and *Xiphydria camelus*, but not in other Symphyta^23^. On the other hand, the blue photoreceptor seems to be missing in all but one species, *T. campestris*. More recent studies assumed that the blue photoreceptor was present but not found experimentally^9,16^. To date, the number of visual opsins and what mechanisms could have driven photoreceptor loss and gain in the Symphyta are unknown.

The biology of the diurnal woodwasp *S. noctilio* (Symphyta) suggests an important role of vision. There is a strong sexual dimorphism, where males are black with an orange abdomen and females are completely black with steel blue iridescence (from a human perspective). Field studies have reported that males aggregate at the top of pine trees to form leks and females are attracted to these for mating^25–27^. A male specific putative pheromone released from the sexually dimorphic hind legs has been described^28^. We refer to this as a putative pheromone because although attraction was observed in the lab^29^, it was inactive in field trials^30^ suggesting that other sensory modalities than olfaction could play an important role in mate attraction. In addition, a study of flight behaviour showed that females exhibit a higher altitude when males are present compared to when males are absent^31^. A field trapping study observed that simulated leks, where traps were baited with dead males, increased the number of females captured in traps, potentially due to visual signals^32^. Finally, the addition of UV light greatly increased the number of *S. noctilio* captured in different trap types^33^. Despite these indications of the importance of visual stimuli on the behaviour of the woodwasp, the visual ecology of *S. noctilio* is poorly understood.

The evidence so far suggests an important role for (colour) vision and a potential lack of a SW opsin in the Siricidae. Due to its phylogenetic position between the potentially tetrachromatic *Tenthredo campestris* and the trichromatic Apocrita^9,23^, *S. noctilio* is a promising model to better understand the evolution of colour vision in basal Hymenoptera. In this study, we (i) determined the spectral sensitivity of the compound eyes of *S. noctilio* by means of electroretinography, (ii) characterised the diversity of visual opsin genes using genomic tools and (iii) analysed opsin expression in the compound eyes and ocelli of *S. noctilio*.

## Results

### ERG

**Figure 1:**
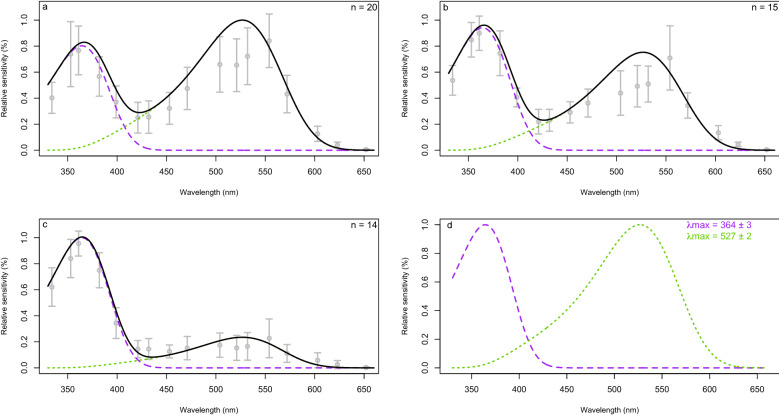
Spectral sensitivity of the compound eyes of *S. noctilio* determined by ERG after (**a**) dark adaptation (n = 10 males and 10 females), (**b**) dim green light adaptation (n = 7 males and 8 females), and (**c**) bright green light adaption (n = 7 males and 7 females). Normalized spectral sensitivity of the two types of receptors (**d**). Grey circles represent the relative sensitivity (%) measured from ERGs (mean ± standard deviation). Dashed (purple) and dotted (green) lines indicate the ∆(Qi) of the UV and LW receptors, respectively, under each adaptation. The black line shows the sum of the relative contribution of the UV and LW receptor model multiplied by the respective scaling factors for each adaptations.

For each light environment, no difference between male and female spectral sensitivity was detected (Kruskal–Wallis test > 0.05 for both LW1 and UV). Therefore, data from both sexes were pooled and averaged for curve fitting. Ten females and ten males were measured under dark adaptation (Fig. 1a). The λ_max_ of the LW photoreceptor was determined to be λ_max_ = 527 ± 2 nm. Eight females and seven males were measured under dim green light adaptation (551 nm at 7 × 10^12^ quanta/s/cm^2^) (Fig. 1b). Seven females and seven males were measured under strong green light adaptation (551 nm at 7 × 10^15^ quanta/s/cm^2^) (Fig. 1c). These recordings were used to determine the λ_max_ of the UV photoreceptors with minimal contribution of the LW receptors. The λ_max_ of the UV photoreceptor under strong green light adaptation was found to be λ_max_ = 364 ± 3 nm (residual standard error = 0.121, Student t-test p-value < 2 × 10^–16^). No evidence of a potential blue photoreceptor was visible when both the UV- and LW-light adapted models (Fig. 1, solid black lines) were fit to the responses of the eyes of the three adapted states.

### Genetic analyses

Three putative visual opsin genes were found in the genome of *S. noctilio*. The BLAST analyses (Table S3) revealed that genes on the scaffolds 6, 7 and 692 of the *S. noctilio* genome assembly were the best matches for the UV, LW1 and LW2 opsin genes from both *Apis mellifera* (Apidae) and *Orussus abietinus* (Orussidae). After curation, the genes in the genomic sequences consisted of 1658, 2549 and 2381 nucleotides, respectively, for the putative UV, LW1 and LW2 opsins. The peptide sequences were made of 371 amino acids including five exons for the putative UV opsin, 383 amino acids including five exons for the putative LW1 opsin and 400 amino acids including eight exons for the putative LW2 opsin. Visual opsin genes identified in this study were deposited in the NCBI library under the accession numbers MW340972–MW340974.

A total of 209 visual opsin sequences were found in 78 hymenopteran species including 77 LW1, 43 LW2, 43 SW, and 46 UV opsins (Table S4). In 42 species, the complete pool of visual opsins were located because the genome was available or all the opsins could be extracted. These 42 species show few variations within opsin groups, where 41 LW1, 40 LW2, 41 SW and 42 UV were found. All species possess a copy of the UV opsin. The European woodwasp, *S. noctilio*, is the only species lacking the SW opsin. The LW2 opsin was not found in two species of ants, *Cardiocondyla obscurior* and *Cerapachys biroi*. One species of ant, *Camponotus rufipes*, did not have a copy of the LW1 opsin. The remaining 36 species thought to have an incomplete pool of opsins includes 28 Myrmecia species, *Nothoyrmecia macrops, Osmia rufa*, *Diadasia afflicta*, *Diadasia rinconis*, *Camponotus atriceps* and *Cataglyphis bombycinus*, from which only the LW opsins were extracted, and *Tenthredo koehleri* and *Chrysis viridula*, where the LW2 opsin was not found.

**Figure 2:**
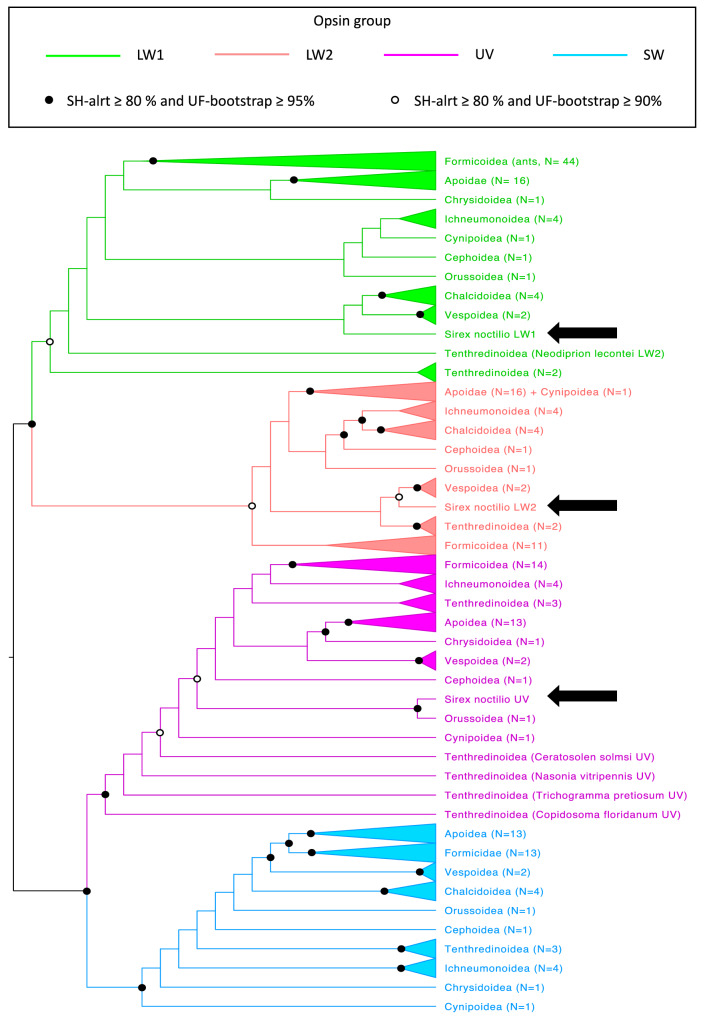
Hymenopteran maximum likelihood tree of LW1 (green), LW2 (red), UV (purple) and SW (blue) opsins amino acid sequences. SH-aLRT (out of 10,000 replicates) ≥ 80% and bootstrap value (out of 10,000 replicates) ≥ 90% (open circle) or ≥ 95% (black filled circle) are shown. Position of the visual opsin genes found in *S. noctilio* were indicated with a black arrow. Accession numbers and curated sequences are available (Table S1).

The phylogenetic analysis (Fig. 2) of the visual opsin amino acids sequences in Hymenoptera resolved four clearly separated clades representing the formerly described opsin groups (SH-aLRT ≥ 80% and UFboot ≥ 95%). The UV opsins grouped sister to the SW opsins. The LW 1 and LW2 genes fall into monophyletic clades that are sister to one another.

The translated sequences of the three putative visual opsin genes in *S. noctilio* fall within three of the four groups. The putative visual opsin sequences found on scaffold 6, 7 and 692, respectively, fall within the UV, LW1 and LW2 opsin groups. The UV opsin sequence of *S. noctilio* groups with *O. abietinus* (Symphyta), and is closely related to the *Cephus cinctus* (Symphyta) UV opsin sequence. The LW1 opsin sequence of *S. noctilio* groups the closest with the LW1 opsin sequence of the Vespoidea (Apocrita) and Chalcidoidea (Apocrita) species. The LW2 opsin sequence in *S. noctilio* groups most closely with the LW2 opsin sequence of the Tenthredinoidea (Symphyta) species.

**Figure 3:**
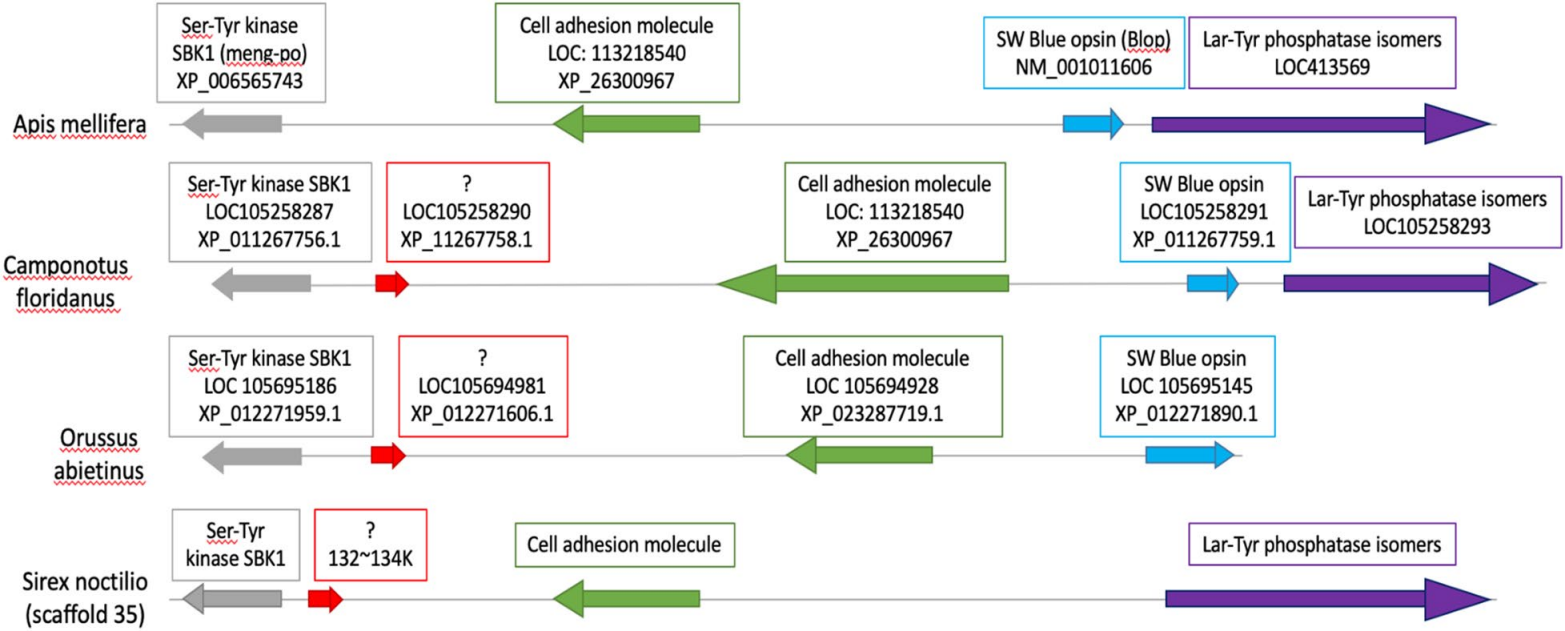
Representation of the SW flanking gene in three hymenopterans. Similar colour corresponds to orthologs. The SW opsin gene in *A. mellifera*, *O. abietinus* and *C. floridanus* is flanked by the Cell adhesion molecule (green) gene and the Lar-Tyr phosphatase (purple) genes, also found in *S. noctilio.*

No SW opsin gene was found in the genome of *S. noctilio* (Fig. 3). No high-scoring matches were obtained from the BLAST search analyses of the SW opsin genes from other Hymenoptera in the genome of *S. noctilio*. Local BLAST searches using the genes flanking the SW opsin gene in other Hymenoptera (including *A. mellifera*, *O. abietinus* and *C. floridanus*) as queries against the *S. noctilio* genome indicated that the *S. noctilio* orthologues of these flanking genes are located on scaffold 35 of the genome assembly. Gene regions coding for Lar-Tyr phosphatase genes were found to be present downstream of the SW opsin gene in *A. mellifera* and *C. floridanus*. No Lar-Tyr phosphatase genes were found in *O. abietinus* likely because the available assembled scaffold ended after the SW opsin gene. Two genes were found upstream of the SW opsin gene in *A. mellifera*, *O. abietinus* and *C. floridanus*. These two genes code for a cell adhesion molecule and a Ser-Tyr kinase SBK1 gene, and both could be identified in *S. noctilio.* In addition, a gene coding for an unknown protein located between the cell adhesion molecule and the Ser-Tyr kinase SBK1 genes was observed in *O. abietinus*, *C. floridanum* and *S. noctilio*. However, the SW opsin gene in *S. noctilio* was not found between the Lar-Tyr phosphatase genes on one side and the cell adhesion molecule on the other side, as in other hymenopterans. No sequence similarities to other hymenopteran species could be identified when performing BLAST analyses of the genomic section where the SW opsin gene was expected to be found in *S. noctilio*. Assessment of genome sequence reads mapped back to the scaffold where the SW opsin was expected to be found indicated that the absence of this gene cannot be explained by incorrect assembly of the *S. noctilio* genome.

### RT-PCR analyses

Gene expression analyses show that the LW1, LW2 and UV genes are differentially expressed in the compound eyes and ocelli (Fig. 1, Supplementary material). The *LW1* gene was expressed in the compound eyes of both male and female woodwasps. In contrast, the *LW2* gene was expressed only in the ocelli of both sexes. The UV gene was the only opsin expressed in both the compound eye and the ocelli. Sequencing analyses of the expressed PCR product confirmed that the expected transcripts were amplified (Fig. 2, Supplementary material).

## Discussion

Our ERG bioassays and RT-PCR analyses showed two photoreceptors and two visual opsins expressed in the compound eyes of *S. noctilio*. The photoreceptors in the compound eyes revealed peak maxima at λ_max_ = 527 ± 2 nm and 364 ± 3 nm, respectively. Consistent with previous studies, *LW1* was found to be expressed in the compound eyes, *LW2* was found to be ocelli specific and the *UV* opsin was found to be expressed in both compound eyes and ocelli as has been shown in bees^34^. The peak maxima in *S. noctilio* are similar to previous intracellular recording studies on Symphyta (Siricoidea, Xiphidrioidea and Tenhredinoidea), where a blue photoreceptor was also not found^23^. Later studies speculated that the blue photoreceptor was present but missed, or that the expression levels were much lower than for LW-sensitive photoreceptors and therefore not detected^9^.

We could not find any evidence of a SW opsin (pseudo-)gene in the genome of *S. noctilio*, suggesting that the complete loss of the SW opsin likely did not occur recently in *S. noctilio,* but at a higher phylogenetic level. We thus suggest that earlier studies may have failed to detect a blue photoreceptor not because of low expression levels, but rather because the SW opsin coding for a blue light-sensitive photoreceptor has been lost by a common ancestor. The latest phylogeny for the Hymenoptera suggests that Siricoidea and Xiphidrioidea form a monophyletic group^35^. A blue photoreceptor was also not found in the sawfly *Urocerus gigas* (Siricoidea) and in the woodwasp *X. camelus* (Xiphidrioidea)^23^. Based on our data and previous intracellular recordings^23^, it is possible that the loss of SW opsin gene that gave rise to a blue-light sensitive photoreceptor already occurred in the common ancestor of both families. Alternatively, the loss of the blue receptor has occurred multiple times within the Symphyta.

The loss of the SW opsin gene and related blue photoreceptor in *S. noctilio* might be explained by its ecology and specific feeding habits*.* Adults of *S. noctilio* and other Siricidae do not feed, and thus have no need for flower recognition or detection of other food sources^36,37^. The adult lives for only a few days^38^ and only needs to find mates and oviposition sites. Selective pressure could have driven the loss of the SW opsin gene and blue photoreceptor if not needed anymore. Investigating the opsin gene repertoire of species with feeding and non-feeding adults in the future will help to understand the evolution of visual opsin genes in Symphyta.

Another possible scenario could be that a nocturnal ancestor lost the SW opsin inherited in Siricoidea and other Symphyta. For example, in the superorder Neuropteroidea^39^, the American cockroach *Periplaneta Americana*^40^ and the praying mantis *Tenoda sinensi*^41^, the loss of SW opsin is linked to a common nocturnal ancestor in each of the clades. In contrast, beetles restored a blue photoreceptor by the duplication and tuning of a second UV opsin gene^39,42^. The restoration of a functional blue light-sensitive photoreceptor might be an advantage in diurnal pollinator insects such as beetles. We saw no evidence for a duplication of a UV opsin that could have restored a functional blue photoreceptor in *S. noctilio*. In future studies, genomic comparisons of the SW opsin genes of Symphyta species from different families could ascertain if the SW opsin was lost by a common ancestor or in *S. noctilio* only.

Some other hymenopterans also lack a functional blue photoreceptor. In the ant *Camponotus rufipes*, for example, a SW opsin is present in the genome, but its level of expression is much lower than the level of UV and LW opsins^43^. Behavioural colour vision studies in the closely related species, *C. blandus*, also showed that the animals are dichromates, with only a UV and green-sensitive photoreceptor^44^. It was speculated that the loss of a functional blue photoreceptor in some ants could be linked to ecology and a limited use of landmarks for navigation^45^. There is no evidence for path integration or strong navigation skills in *S. noctilio*, and forest landmarks are relatively poor in colours. From this perspective, a landmark-poor environment and a limited need for orientation could have driven the loss of functional blue photoreceptor in *S. noctilio* or its ancestor.

No red photoreceptor was found in our ERG analyses. Intracellular recordings on two species of Tenthredinoidea and one species of Xiphidrioidea suggested that these families possess a red-sensitive photoreceptor^23^. In the same study, a red photoreceptor was not found in the one species of Siricoidea tested (*U. gigas*). The red photoreceptor was likely lost in the Siricoidea and more recent families, but might still be present in earlier diverged families such as Xiphidrioidea and Tenhredinoidea. In-situ hybridisations and genetic comparisons could ascertain if the red photoreceptor found in Symphyta is based on an additional opsin gene, or derived from screening pigments that shift the sensitivity of two photoreceptors expressing the same opsin^14^.

The broad sensitivity of the green photoreceptor in our ERG analyses and the amplified LW2 band visible in the RT-PCR are unlikely due to a second green photoreceptor. A very similar absorption spectrum of the LW1 opsin was found if only the ERG data from the red end of the spectrum (550 to 650 nm) were used instead of the entire dataset (data not shown). Thus, the large absorption spectrum is most likely due to noisy data than differently tuned green photoreceptors in this part of the compound eyes. The additional band visible in the RT-PCR gel of the amplified LW2 gene in the compound eyes is likely caused by DNA contamination due to the high similarity of the amplicon and the DNA control with intron (see Fig. 1, supplementary material). However, future transcriptomic analyses of the compound eyes could ascertain if variants of the LW1 or LW2 homologs are expressed in the compound eyes.

The expression of two opsins in the ocelli as observed in *S. noctilio* is well conserved in flying insects (e.g. Orthoptera^46^, Odonata^1^ and Hymenoptera (bees)^47^). In Hymenoptera, the presence of a LW ocelli-specific opsin has been confirmed in bees^21,34^, fig wasps^24^, and in the Siricidae (this study). In other insects, the UV photoreceptor was found but the corresponding opsin could not be characterised (praying mantis^41^, moth^48^ and ants^49^). In some ants, the ocelli have been linked to celestial compass orientation^50^, where only a UV photoreceptor may be sufficient for this purpose. In contrast, for most flying hymenopterans, in contrast (and possibly flying insects in general), two opsins expressed in the ocelli seem to provide an advantage for flight stabilisation. The UV-green contrast together with the triangular arrangement of the ocelli provides a solid skyline delimitation^51^. On flight mill experiments *S. noctilio* has been observed to fly as much as 19–49 km in a 24 h period^52,53^. An ocellar system that enhances flight stabilisation is consistent with this high dispersal capacity.

Our study characterized the visual system of *S. noctilio* at the genetic and physiological level and lays the foundation for further work on the visual systems of basal Hymenoptera. To our knowledge, *S. noctilio* is the first confirmed case of lack of the SW opsin gene and blue light-sensitive photoreceptor (i.e., genetic and physiological evidence) in the Hymenoptera. Future studies should be conducted to ascertain if *S. noctilio* is a dichromatic species (including e.g. behavioural tests^44^, neuronal interactions^10^). Genetic comparison of species from different families of Symphyta and with different feeding habits will determine if the loss of the SW opsin gene occurred only in *S. noctilio,* or already at a higher phylogenetic level. Studying opsin evolution in Symphyta, and particularly the loss and gain of blue- and red-light sensitive photoreceptors, offers exciting opportunities to understand the ecological relevance of gain and loss of visual opsin genes and its link with di-, tri- and tetra-chromacy in closely related families of insects.

## Materials and methods

### Insects

Pine logs infested with *S. noctilio* were collected from a pine plantation in Knysna (South Africa) (n = 133). Trees with characteristic symptoms of infestation, such as brown needles and fresh resin droplets oozing from the bark from oviposition sites were selected and cut into logs. Logs were stored in an insectarium at 20 °C with ambient humidity and a photoperiod of 12/12 h L/D. After emergence, insects were collected and stored in a fridge at 12 °C for later use. Before any analyses were performed, insects were left under artificial light for 30 min at room temperature. The use of insect and plant materials in this study complies with international, national and/or institutional guidelines.

### Electroretinographical recordings (ERG)

To reduce external noise, recordings were conducted in a grounded Faraday cage painted completely black. Live, intact animals were fixed in a custom-made Plexiglas holder using dental wax. Antennae were carefully fixed to ensure they did not obstruct the measured compound eye. The reference electrode (25 µm silver wire) was inserted into the head capsule behind the compound eye. Before inserting the recording electrode, a small hole was drilled into the cuticle of the compound eye using a minute pin. Recording electrodes were glass capillaries pulled with a DMZ-Universal Puller (DMZ-Universal Puller, Zeitz-Instruments, Germany) filled with 1 molar Potassium-Chloride solution. To be consistent among insects, recording electrodes were always placed carefully at the centre of the compound eye just under the cuticle surface. The signal was 10× amplified (Neuroprobe Amplifier Model 1600, A-m System Inc. Sequim, USA) and Bandpass filtered 0.1–150 Hz using a dual variable filter (VBF 8, Kemo Inc., Greenville, USA). The filtered analogue signal was digitized using an analogue–digital receiver (Lap-Trax 4/16, World Precision Instruments, USA) to be visualized and recorded with the computer software LabScribe (LabScribe Version 3.010800, iWorx Systems Inc., USA).

### ERG: light stimulation

A xenon arc lamp (Abet Technologies Inc., Model LS-150-Xe SN 127, Milford, USA) was used to produce a full daylight spectrum with a spectral range from 320 to 820 nm. Optical band pass filters (Edmund Optics Inc., Barrington, USA) with a 20 nm full width-half maximum, produced the monochromatic light stimuli tested. For each stimulus, light intensity was adjusted to 1.04 ± 0.07 × 10^14^ photons/cm^2^ × s using a motor driven continuously variable neutral density filter (Nanotec-Munich, Model ST2818L1006B, Munich, Germany) controlled by an Arduino board. This value was based on the maximum light intensity passing through the most selective monochromatic filter we could obtain. Two motor driven wheels (Lambda 10-2, Model LB10-2, Shutter Instrument, Novato, USA) equipped with the different band pass filters were positioned successively into the light beam. The first one included a shutter to produce light flashes of 100 ms. The Arduino, the shutter and the filter wheels could be controlled using a custom-made MatLab script (The MathWorks Inc., MatLab R2014a, Version 8.3.0.532). After the light has passed the filters, it was guided through an optical quartz fibre (500 µm diameter) placed 1 cm in front of the eye so the light spot was larger than the insect head.

Insects were first dark-adapted for 15 min prior to the experiment. Before and after each series of spectral measurements, eight increasing intensities of white light were flashed for 100 ms on the insect eye. The range of white light intensities was generated by passing the light through a combination of eight positions of the neutral grey filter, which created intensities between 5.97 × 10^12^ to 4.95 × 10^16^ photons/cm^2^ × s. After the last white light flash, insects were given 15 min to readapt to ambient light conditions before the first round of spectral measurements started. For each series of spectral measurement, two rounds of 17 monochromatic lights (334, 352, 358, 382, 398, 418, 431, 449, 468, 501, 519, 529, 550, 569, 599, 620 and 649 nm) were flashed in random order for 100 ms with 1 min between each flash of light. One minute after the first round, a second round of the same 17 monochromatic lights were flashed in a random order. This procedure was repeated for three light adaptation levels. When the last white flash from the first experiment was finished, insects were left for 15 min under a constant dim green light (551 nm at 7 × 10^12^ quanta/s/cm^2^) placed 1 cm in front of the eye and the procedure was repeated. The same procedure was used a third time under a constant strong green light (551 nm at 7 × 10^15^ quanta/s/cm^2^). Adaptation lights were placed to pass through the light guide. Individuals were excluded from the dataset if no depolarisation was observed to the brightest white light.

### ERG analyses

For each individual and under each light adaptation, a V-log(I) curve was computed. The depolarisation for each white light intensity flashed before and after the spectral measurements were averaged and a logarithmic model was used to create a V-log(I) curve. In order to start at log(0), the different intensities were log(I) transformed and the log(I) of the weakest value was subtracted from each white light intensity. The amplitude signal was transformed into the equivalent intensities log(I) for each monochromatic wavelength tested with the V-log(I) curve. The sensitivity (S) for each wavelength tested was computed using the method described in Telles et al*.*^54^ with the following equation:

$$S(\lambda )={10}^{(\mathrm{log}\left(I\right)-\mathrm{log}({I}_{max})}$$

where log(I) is the equivalent intensity for each response, and log(I_max_) is the equivalent intensity of the highest response for each series. Sensitivity values were averaged for each sex under the same light adaptations. The difference in quantum catch for the receptor i (∆(Qi)) and a colour stimulus was calculated with the equation:

$$ \Delta \left( {{\text{Qi}}} \right)  = {\text{ki }} \times {\text{Ri}}({{\lambda}}), $$

where Ri(λ) is the sensitivity of the receptor i for the wavelength λ, and ki an arbitrary scaling factor for the photoreceptor i for each light adaptation^55^. The Ki for the UV peak under strong green light adaptation and for the LW1 photoreceptor under dark adaptation was defined as 1 since these adaptations are maximising the sensitivity of each photoreceptor. For each light adaptation, the sensitivity of the wavelength measured (Ri measured) at 364 nm for the UV and 527 nm for the LW1 was divided by the Ri measured under strong green light and dark adaptation, respectively.

### Statistical analyses

The adapted Stavenga 2 parameter opsin template^56^ was used to determine the λ_max_ of each photoreceptor. The averaged sensitivity value for each receptor was fitted with the nlsLM function from the “minpack.lm” package in R (https://cran.r-project.org) into the different templates with the corresponding constant for each model^56^. Models were compared and the residual standard error and P-value were used as a measure to choose the best model fitting the data.

### Genetic analyses

Genomic coding sequences (CDS) of the visual opsin genes LW1, LW2, UV and SW of *A. mellifera* (*Lop1*, *Lop2*, *Uvop* and *Blop*) and *O. abietinus* were used to perform a local BLASTp and BLASTn^57^ search against the genome assembly and annotation of *S. noctilio* (Alisa Postma et al*.* unpublished data) using CLC Main Workbench V7.7.3 (http://www.clcbio.com). Sequences with an E-value < 10^–20^ were retained for further analyses. The Apollo Genome Annotation and Curation tool^58^ was used to curate the identified gene models.

The SW opsin gene was not identified using the above-mentioned BLAST searches; therefore, a targeted search for the SW opsin gene in *S. noctilio* was conducted. Local BLASTn, BLASTp and tBLASTn searches were performed using the SW opsin nucleotide and amino acid sequences from *A. mellifera*, *O. abietinus* and *Camponotus floridanus* species as queries against the genome assembly (nucleotide sequence) and the annotation (protein sequences) of *S. noctilio*. The flanking genes of the SW opsin gene from the genome of *A. mellifera*, *O. abietinus* and *C. floridanus* were identified and used to perform local BLAST searches against the *S. noctilio* genome. The corresponding flanking genes in *S. noctilio* were annotated. The section in scaffold 35 surrounded by the flanking genes in *S. noctilio* where the SW opsin was found in other insects was used to perform local BLAST analyses on NCBI including all available hymenopteran genomes. In order to assess whether the absence of the SW opsin gene in the *S. noctilio* assembly could be ascribed to errors/misassembly of the genome sequence, the raw genomic sequencing reads used to generate the genome assembly of *S. noctilio* were mapped back to scaffold 35 from the *S. noctilio* genome using the Burrows-Wheeler Aligner (BWA)^59^. The resulting alignments were analysed and visualised using the Integrative Genomics Viewer (IGV)^60^.

Hymenopteran visual opsin DNA, RNA and protein sequences were obtained from the literature and online resources including GenBank^61^, OrthoDB^62^ and i5K^63^. For these sequences, the gene predictor Augustus^64^ was used to remove potential introns and to translate DNA sequences into amino acid sequences. RNA sequences were translated into amino acid sequences via MEGA7^65^. Amino acid sequences were aligned using MAFFT^66^ under default parameters. Data were visually curated after alignment. Accession numbers of the hymenopteran sequences used for the phylogenetic reconstruction and aligned curated sequences are available (Table S1).

Phylogenetic reconstruction was performed in IQTree v1.4.4^67^. The most likely amino acid substitution model was found to be the LG + F + I + G4. This model was used to build a Maximum Likelihood tree with 10,000 ultrafast bootstrap iterations^68^ and 10,000 SH-like approximate likelihood ratio tests^69^ were used to assess nodal support. The tree was rooted at the midpoint and visualised in FigTree v1.4.3 (http://tree.bio.ed.ac.uk/software/figtree/) .

### RT-PCR analyses

RNA was extracted from both male and female *S. noctilio*. The compound eyes and the ocelli were surgically removed using a clean scalpel blade and stored individually in Eppendorf tubes at − 80 °C. The compound eyes and ocelli of ten individuals of each sex were pooled, and a NucleoSpin RNA purification kit (Macherey–Nagel) was used to extract and purify the RNA. The quantity and quality of RNA was assessed by means of a nanodrop and a 2% electrophorese gel, respectively. The RNA was then transformed into cDNA via a cDNA synthesis kit (SensiFAST).

Specific primers for *S. noctilio* were designed to amplify the LW1, LW2 and UV opsin genes in the Genscript primer design software (https://www.genscript.com/tools/real-time-pcr-taqman-primer-design-tool) using default parameter values (Table S2). Primers were designed with at least one intron present between the forward and reverse primer so that the amplified RNA product could be differentiated from accidentally amplified DNA contamination. Reverse transcription polymerase chain reactions (RT-PCR) were performed for all samples and primers as follows: denaturation at 95 °C for 5 min, followed by 40 cycles of 94 °C for 30 s, 49 °C for 30 s, 72 °C for 1 min, then 7 min at 72 °C. The RT-PCR products were run on 2% agarose gel at 110 V, 400 mA for 30 min. The RT-PCR products were prepared for sequencing when the genes were thought to be expressed. A PCR clean-up was performed by adding 8 µL of Exo-SAP to the PCR product and put at 37 °C for 15 min and 80 °C for 15 min. A sequencing PCR was performed by adding 6.4 µL of dH_2_O, 2.1 µL of sequencing buffer, 0.5 µL of BigDye, 1 µL of primer and 2 µL of cleaned PCR product. The PCR thermocycling profile used was 27 cycles of 96 °C for 10 s, 55 °C for 15 s, 60 °C for 4 min. Samples were subsequently washed and precipitated for sequencing. Samples were cleaned with 50 µL of 100% EtOH, 2 µL of NaOAc and 8 µL of dH_2_O and centrifuged at 13,400 rpm and 4 °C for 30 min. The supernatant was removed and 150 µL of 70% EtOH was added and the mixture centrifuged at 13,400 rpm and 4 °C for 10 min. This step was repeated a second time. The supernatant was removed and tubes were left open under the fume hood overnight to dry. Dried samples were sent for Sanger sequencing at FABI, University of Pretoria (South Africa). Sequencing results were manually curated in CLC Main workbench. Base calling conflicts were resolved by selecting the peak with the highest relative fluorescence units. Noise and contamination data under 4000 relative fluorescence units were eliminated. Sequences were aligned with the corresponding genomic sequence under default parameters.

## Supplementary Information

Supplementary Information.

## Data Availability

https://dataverse.harvard.edu/dataset.xhtml?persistentId=doi:10.7910/DVN/HCNMW4.
