# Supplementary material for: Evidence for UV-green dichromacy in the basal hymenopteran Sirex noctilio (Siricidae)
Source: Sci Rep. 2021 Aug 2;11:15601. doi: 10.1038/s41598-021-95107-2 (PMC8329207; doi:10.1038/s41598-021-95107-2)

**Evidence for UV-green dichromacy in the basal hymenopteran *Sirex noctilio* (Siricidae)**

**Quentin Guignard, Johannes Spaethe, Bernard Slippers, Martin Strube-Bloss, Jeremy D. Allison**

**Supplementary material caption**

Table S1: Accession number of the opsin genes used in this study. Curated and aligned sequences are available at https://dataverse.harvard.edu/dataset.xhtml?persistentId=doi:10.7910/DVN/HCNMW4

Table S2: Details of the primers used for RNA extraction for the LW1, LW2 and UV opsins of *S. noctilio.*

Table S3: BLAST search results of the different visual opsins from *A. mellifera* and *O. abietinus* found in the genome of *S. noctilio*. E-values above 10^-20^ were found in scaffold 6, 7 and 692 respectively matching the UV, LW1 and LW2 opsins of *A. mellifera* and *O. abietinus*.

Table S4*:* Taxonomy (based on ^35^) and number of opsins found for each Hymenopteran species used for the phylogenetic analyses.

Figure S1: PCR products from the cDNA extract from the compound eyes (CE) and ocelli (O) in *Sirex noctilio*. The LW1 (top), LW2 (middle) and UV (bottom) PCR were done independently. The amplified LW1 product visible in the O of males and females and in the DNA (faint band indicated by a black arrow) corresponds to the 324 base pair (bp) amplicon with the intron. The visible band in the CE corresponds to the 151 bp amplicon amplified without the intron, indicating that the LW1 gene is expressed in the CE of both males and females. The amplified LW2 product visible in the DNA control and in the CE of male and female *S. noctilio* correspond to the expected size of around 500 bp with the intron, and is most likely DNA contamination in the CE. The intron of the LW2 gene in the genome assembly contained ambiguous sequences and is likely incomplete, which makes the exact size difficult to determine. Two sets of bands are visible in the O of both males and females. The bottom bands correspond to the 296 bp amplicon without the intron and indicates that the LW2 gene is expressed in the ocelli of both males and females. The top bands correspond to the approximately 500 bp amplicon with the intron and is most likely DNA contamination. The four bands visible for the UV opsin gene in male, female, CE and O correspond to the amplified 163 bp amplicon without the intron in both CE and O. The visible band in the DNA control correspond to the 493 bp amplicon amplified with the intron. Primers were designed on two distinct exons, so expressed genes migrate further than the amplified contaminated DNA, which contains at least one intron (see Table 2 in supplementary material for more details on the primers used and Figure 2 in supplementary material for the sequencing result of the expressed opsins). We show the original gel pictures which were not modified. Blank samples are negative controls without DNA or RNA, DNA samples are positive controls containing DNA extracted from *S. noctilio*. Bands visible at the bottom of the UV gene lane correspond to the amplification of non-specific amplicons.

Figure S2: Alignment of the sequenced LW1, LW2 and UV cDNA with their corresponding genomic sequences (including introns) in *S. noctilio*. From top to bottom: expressed LW1 gene aligned with the opsin found in scaffold 7, expressed LW2 gene aligned with the opsin found in scaffold 692, expressed UV gene aligned with the opsin found in scaffold 6

Table S1 Supplementary material

| Species name | Gene name | Accession number | Database |
| --- | --- | --- | --- |
| *Acromyrmex echinatior* | UV1 | AECH14675 | OrthoDB |
| *Acromyrmex echinatior* | LWS | AECH18073 | OrthoDB |
| *Acromyrmex echinatior* | LWS | AECH18074 | OrthoDB |
| *Acromyrmex echinatior* | SW1 | AECH19964 | OrthoDB |
| *Apis cerana* | LW1 | ACSNU07734T0 | OrthoDB |
| *Apis cerana* | LW2 | ACSNU07733T0 | OrthoDB |
| *Apis cerana* | SW1 | ACSNU05647T0 | OrthoDB |
| *Apis cerana* | UV1 | AB355816.1 | GenBank |
| *Apis dorsata* | UV1 | 51584357 | OrthoDB |
| *Apis dorsata* | LWS | 51569490 | OrthoDB |
| *Apis dorsata* | LWS | 51569495 | OrthoDB |
| *Apis dorsata* | SW1 | 51578434 | OrthoDB |
| *Apis florea* | UV1 | 2481023892 | OrthoDB |
| *Apis florea* | LW1 | 2379349636 | OrthoDB |
| *Apis florea* | LW1 | 2379349700 | OrthoDB |
| *Apis florea* | SW1 | 2478935732 | OrthoDB |
| *Apis mellifera* | Lop1 | NM_001011639 | GenBank |
| *Apis mellifera* | Lop2 | NM_001077825 | GenBank |
| *Apis mellifera* | Blop | NM_001011606 | GenBank |
| *Apis mellifera* | Uvop | NM_001011605 | GenBank |
| *Athalia rosae* | UV1 | AROS003931 | OrthoDB |
| *Athalia rosae* | LW2 | XP_012261693.1 | GenBank |
| *Athalia rosae* | LW1 | AROS000476 | OrthoDB |
| *Athalia rosae* | SW1 | AROS010079 | OrthoDB |
| *Atta cephalotes* | UV1 | ACEP24081 | OrthoDB |
| *Atta cephalotes* | LWS | ACEP19443 | OrthoDB |
| *Atta cephalotes* | LWS | ACEP19445 | OrthoDB |
| *Atta cephalotes* | SW1 | ACEP24672 | OrthoDB |
| *Bombus impatiens* | UV1 | AY655163.1 | GenBank |
| *Bombus impatiens* | LWRh1 | AY485302.1 | GenBank |
| *Bombus impatiens* | LWRh2 | AY485306.1 | GenBank |
| *Bombus impatiens* | SW1 | Bimp_2.0 | OrthoDB |
| *Bombus terrestris* | UV1 | 15202080 | OrthoDB |
| *Bombus terrestris* | LW1 | 15205607 | OrthoDB |
| *Bombus terrestris* | LW1 | 15205608 | OrthoDB |
| *Bombus terrestris* | SW1 | 15207536 | OrthoDB |
| *Camponotus atriceps* | LW1 | Q17292.1 | GenBank |
| *Camponotus atriceps* | UV1 | AAC05092.1 | GenBank |
| *Camponotus floridanus* | UV1 | CFLO14976 | OrthoDB |
| *Camponotus floridanus* | LW1 | CFLO13350 | OrthoDB |
| *Camponotus floridanus* | LW1 | CFLO13353 | OrthoDB |
| *Camponotus floridanus* | SW1 | CFLO18506 | OrthoDB |
| *Camponotus rufipes* | LW2 | KT261378 | GenBank |
| *Camponotus rufipes* | UV | KT261377 | GenBank |
| *Camponotus rufipes* | SWS | KT261376.1 | GenBank |
| *Cardiocondyla obscurior* | UV1 | Cobs_00666 | OrthoDB |
| *Cardiocondyla obscurior* | LW1 | Cobs_07608 | OrthoDB |
| *Cardiocondyla obscurior* | SWS | Cobs_14313 | OrthoDB |
| *Cataglyphis bombycinus* | LW1 | Q17296.1 | GenBank |
| *Cataglyphis bombycinus* | UV1 | AAC05091.1 | GenBank |
| *Cephus cinctus* | UV1 | CCINC:0000f9 | OrthoDB |
| *Cephus cinctus* | LW1 | CCINC:000859 | OrthoDB |
| *Cephus cinctus* | LW1 | CCINC:00291a | OrthoDB |
| *Cephus cinctus* | SW1 | CCINC:0006bf | OrthoDB |
| *Cerapachys biroi* | UV1 | EZA56359.1 | OrthoDB |
| *Cerapachys biroi* | LW1 | EZA56726.1 | OrthoDB |
| *Cerapachys biroi* | SW1 | EZA48811.1 | OrthoDB |
| *Ceratosolen solmsi* | LW1 | JX402130 | OrthoDB |
| *Ceratosolen solmsi* | LW2 | JX402131 | OrthoDB |
| *Ceratosolen solmsi* | SWS | JX402132 | OrthoDB |
| *Ceratosolen solmsi* | UVS | JX402133 | OrthoDB |
| *Chrysis viridula* | UVS | APY20525.1 | GenBank |
| *Chrysis viridula* | SWS | APY20524.1 | GenBank |
| *Chrysis viridula* | LWS | APY20523.1 | GenBank |
| *Copidosoma floridanum* | LW1 | CFLO011095 | OrthoDB |
| *Copidosoma floridanum* | SW1 | CFLO009114 | OrthoDB |
| *Copidosoma floridanum* | LWS | XP_023246947.1 | GenBank |
| *Copidosoma floridanum* | UVS | XP_014218019.1 | GenBank |
| *Cotesia vestalis* | SWS | APY20529.1 | GenBank |
| *Cotesia vestalis* | LW2 | APY20526.1 | GenBank |
| *Cotesia vestalis* | LW1 | APY20528.1 | GenBank |
| *Cotesia vestalis* | UVS | APY20527.1 | GenBank |
| *Diachasma alloeum* | UV1 | XP_015115701.1 | GenBank |
| *Diachasma alloeum* | LW1 | XP_015110387.1 | GenBank |
| *Diachasma alloeum* | LW2 | XP_015110385.1 | GenBank |
| *Diachasma alloeum* | SW1 | XP_015116842.1 | GenBank |
| *Diadasia afflicta* | LWRh1 | AY485303 | GenBank |
| *Diadasia afflicta* | LWRh2 | AY485308 | GenBank |
| *Diadasia rinconis* | LWRh1 | AY485304 | GenBank |
| *Diadasia rinconis* | LWRh2 | AY485307 | GenBank |
| *Dufourea novaeangliae* | UV1 | DNOVA:0009a7 | OrthoDB |
| *Dufourea novaeangliae* | LW1 | DNOVA:000951 | OrthoDB |
| *Dufourea novaeangliae* | LW1 | DNOVA:000952 | OrthoDB |
| *Dufourea novaeangliae* | SW1 | DNOVA:001188 | OrthoDB |
| *Eufriesea mexicana* | UV1 | EMEXI:000c6e | OrthoDB |
| *Eufriesea mexicana* | LW1 | EMEXI:000aeb | OrthoDB |
| *Eufriesea mexicana* | LW2 | EMEXI:0028d6 | OrthoDB |
| *Eufriesea mexicana* | SW1 | EMEXI:0011f4 | OrthoDB |
| *Euglossa dilemma* | UVS | Edil_09953 | i5k |
| *Euglossa dilemma* | LW1 | Edil_07671 | i5k |
| *Euglossa dilemma* | LW2 | Edil_07551 | i5k |
| *Euglossa dilemma* | SWS | Edil_04295 | i5k |
| *Fopius arisanus* | UV1 | 6707570 | OrthoDB |
| *Fopius arisanus* | LW1 | 6707104 | OrthoDB |
| *Fopius arisanus* | LW2 | 6713149 | OrthoDB |
| *Fopius arisanus* | SW1 | 6712580 | OrthoDB |
| *Habropoda laboriosa* | UV1 | HLABO:002143 | OrthoDB |
| *Habropoda laboriosa* | LW1 | HLABO:002b53 | OrthoDB |
| *Habropoda laboriosa* | LW1 | HLABO:002b54 | OrthoDB |
| *Habropoda laboriosa* | SW1 | HLABO:00190f | OrthoDB |
| *Harpegnathos saltator* | UV1 | HSAL22349 | OrthoDB |
| *Harpegnathos saltator* | LW1 | HSAL18590 | OrthoDB |
| *Harpegnathos saltator* | LW1 | HSAL18599 | OrthoDB |
| *Harpegnathos saltator* | SW1 | EFN81524.1 | GenBank |
| *Lasioglossum albipes* | UV1 | Lalb_06455 | OrthoDB |
| *Lasioglossum albipes* | LW1 | Lalb_06990 | OrthoDB |
| *Lasioglossum albipes* | LW1 | Lalb_06991 | OrthoDB |
| *Lasioglossum albipes* | SW1 | Lalb_13064 | OrthoDB |
| *Leptopilina clavipes* | LW2 | APY20531.1 | GenBank |
| *Leptopilina clavipes* | LW1 | APY20532.1 | GenBank |
| *Leptopilina clavipes* | UVS | APY20533.1 | GenBank |
| *Leptopilina clavipes* | SWS | APY20530.1 | GenBank |
| *Linepithema humile* | UV1 | LH19353 | OrthoDB |
| *Linepithema humile* | LWS | LH12072 | OrthoDB |
| *Linepithema humile* | LWS | LH12077 | OrthoDB |
| *Linepithema humile* | SW1 | LH20507 | OrthoDB |
| *Megachile rotundata* | UV1 | MROTU:002d33 | OrthoDB |
| *Megachile rotundata* | LW1 | MROTU:00205f | OrthoDB |
| *Megachile rotundata* | LW1 | MROTU:002060 | OrthoDB |
| *Megachile rotundata* | SW1 | MROTU:001111 | OrthoDB |
| *Melipona quadrifasciata* | UV1 | MQUAD:001bf8 | OrthoDB |
| *Melipona quadrifasciata* | LW1 | MQUAD:0017d6 | OrthoDB |
| *Melipona quadrifasciata* | LW1 | MQUAD:0017d7 | OrthoDB |
| *Melipona quadrifasciata* | SW1 | MQUAD:001b71 | OrthoDB |
| *Microplitis demolitor* | UV1 | XP_008551484.1 | GenBank |
| *Microplitis demolitor* | LW1 | XP_008557309.1 | GenBank |
| *Microplitis demolitor* | LW2 | XP_008557308.1 | GenBank |
| *Microplitis demolitor* | SW1 | XP_014297257.1 | GenBank |
| *Monomorium pharaonis* | UV1 | 3491790468 | OrthoDB |
| *Monomorium pharaonis* | LW1 | 2754645108 | OrthoDB |
| *Monomorium pharaonis* | LW2 | 2754645156 | OrthoDB |
| *Monomorium pharaonis* | SW1 | 7178555668 | OrthoDB |
| *Myrmecia brevinoda* | LW | BAE44964.1 | GenBank |
| *Myrmecia callima* | LW | BAE44945.1 | GenBank |
| *Myrmecia chasei* | LW | BAE44946.1 | GenBank |
| *Myrmecia chrysogaster* | LW | BAE44952.1 | GenBank |
| *Myrmecia esuriens* | LW | BAE44965.1 | GenBank |
| *Myrmecia eungellensis* | LW | BAE44956.1 | GenBank |
| *Myrmecia fabricii* | LW | BAE44958.1 | GenBank |
| *Myrmecia forceps* | LW | BAE44961.1 | GenBank |
| *Myrmecia formosa* | LW | BAE44955.1 | GenBank |
| *Myrmecia froggatti* | LW | BAE44957.1 | GenBank |
| *Myrmecia fucosa* | LW | BAE44969.1 | GenBank |
| *Myrmecia fulviculis* | LW | BAE44954.1 | GenBank |
| *Myrmecia harderi* | LW | BAE44949.1 | GenBank |
| *Myrmecia loweryi* | LW | BAE44971.1 | GenBank |
| *Myrmecia mandibularis* | LW | BAE44953.1 | GenBank |
| *Myrmecia michaelseni* | LW | BAE44951.1 | GenBank |
| *Myrmecia midas* | LW | BAE44967.1 | GenBank |
| *Myrmecia nigriscapa* | LW | BAE44966.1 | GenBank |
| *Myrmecia nigrocincta* | LW | BAE44973.1 | GenBank |
| *Myrmecia petiolata* | LW | BAE44972.1 | GenBank |
| *Myrmecia picta* | LW | BAE44968.1 | GenBank |
| *Myrmecia pulchra* | LW | BAE44962.1 | GenBank |
| *Myrmecia rowlandi* | LW | BAE44959.1 | GenBank |
| *Myrmecia rufinodis* | LW | BAE44963.1 | GenBank |
| *Myrmecia swalei* | LW | BAE44947.1 | GenBank |
| *Myrmecia tarsata* | LW | BAE44960.1 | GenBank |
| *Myrmecia testaceipes* | LW | BAE44950.1 | GenBank |
| *Myrmecia urens* | LW | BAE44970.1 | GenBank |
| *Nasonia vitripennis* | UV1 | Nasvi2EG000990 | OrthoDB |
| *Nasonia vitripennis* | LW1 | Nasvi2EG003379 | OrthoDB |
| *Nasonia vitripennis* | LW1 | Nasvi2EG003380 | OrthoDB |
| *Nasonia vitripennis* | SW1 | Nasvi2EG004208 | OrthoDB |
| *Neodiprion lecontei* | UV1 | XP_015524256.1 | GenBank |
| *Neodiprion lecontei* | LW1 | XP_015510992.1 | GenBank |
| *Neodiprion lecontei* | LW2 | XP_015510994.1 | GenBank |
| *Neodiprion lecontei* | SW1 | XP_015516920.1 | GenBank |
| *Nothomyrmecia macrops* | LW | BAE44974.1 | GenBank |
| *Orussus abietinus* | UV1 | OABI008892 | OrthoDB |
| *Orussus abietinus* | LW1 | A0A1P8SF62_9HYME | OrthoDB |
| *Orussus abietinus* | LW2 | A0A1P8SF60_9HYME | OrthoDB |
| *Orussus abietinus* | SW1 | A0A1P8SF61_9HYME | OrthoDB |
| *Osmia rufa* | LWRh1 | AY572828 | OrthoDB |
| *Osmia rufa* | LWRh2 | AY572829 | OrthoDB |
| *Pogonomyrmex barbatus* | UV1 | PB27190 | OrthoDB |
| *Pogonomyrmex barbatus* | LW1 | PB12069 | OrthoDB |
| *Pogonomyrmex barbatus* | LW1 | PB27366 | OrthoDB |
| *Pogonomyrmex barbatus* | SW1 | PB20309 | OrthoDB |
| *Poliste canadensis* | LW1 | LOC106789164 | OrthoDB |
| *Poliste canadensis* | LW2 | LOC106789163 | OrthoDB |
| *Poliste canadensis* | UVS | LOC106784314 | OrthoDB |
| *Poliste canadensis* | SWS | LOC106792028 | OrthoDB |
| *Polistes dominula* | UV1 | PdomMRNAr1.2-02384 | OrthoDB |
| *Polistes dominula* | LW1 | PdomMRNAr1.2-04382 | OrthoDB |
| *Polistes dominula* | LW1 | PdomMRNAr1.2-05518 | OrthoDB |
| *Polistes dominula* | SW1 | PdomMRNAr1.2-11069 | OrthoDB |
| *Sirex noctilio* | LW1 | MW340973 | GenBank |
| *Sirex noctilio* | LW2 | MW340972 | GenBank |
| *Sirex noctilio* | UVS | MW340974 | GenBank |
| *Solenopsis invicta* | LW2 | E9IWB4_SOLIN | OrthoDB |
| *Solenopsis invicta* | LW1 | XP_011164467.1 | GenBank |
| *Solenopsis invicta* | Predicted UV | XP_011164985.1 | GenBank |
| *Solenopsis invicta* | SW1 | SINV24180 | OrthoDB |
| *Tenthredo koehleri* | UVS | APY20540.1 | GenBank |
| *Tenthredo koehleri* | LWS | APY20539.1 | GenBank |
| *Tenthredo koehleri* | SWS | APY20538.1 | GenBank |
| *Trichogramma pretiosum* | UV1 | TPRE005600 | OrthoDB |
| *Trichogramma pretiosum* | LW1 | TPRE005398 | OrthoDB |
| *Trichogramma pretiosum* | LW1 | TPRE009663 | OrthoDB |
| *Trichogramma pretiosum* | SW1 | TPRE003427 | OrthoDB |
| *Vollenhovia emeryi* | LW1 | 19044600 | OrthoDB |
| *Vollenhovia emeryi* | LW1 | 19044602 | OrthoDB |
| *Vollenhovia emeryi* | SW1 | 19029100 | OrthoDB |
| *Vollenhovia emeryi* | UVS | 19044702 | OrthoDB |
| *Wasmannia auropunctata* | LW1 | 23041445 | OrthoDB |
| *Wasmannia auropunctata* | LW1 | 23055722 | OrthoDB |
| *Wasmannia auropunctata* | LW1 | 23055723 | OrthoDB |
| *Wasmannia auropunctata* | SW1 | 23064534 | OrthoDB |
| *Wasmannia auropunctata* | UVS | 23049187 | OrthoDB |

Table S2 Supplementary material

| Scaffold | Primer | Sequence | Size of amplicon with intron | Size of amplicon without intron | # of introns |
| --- | --- | --- | --- | --- | --- |
| 6 | UV-reverse | GCCACAACGTACAGGAAACA | 493 | 163 | 1 |
|  | UV-forward | GCCAAATTGTGAGCCACGTA |  |  |  |
| 7 | LW1-Forward | CAAAGGTGCGCTTCTTCGTA | 324 | 151 | 1 |
|  | LW1-Reverse | CGATCTTGAGAGCCAGTCCT |  |  |  |
| 692 | LW2-reverse | CGGCACTAGCAGCTTGATTT | ~500 | 296 | 2 |
|  | LW2-forward | TATCTGGCTGTTCGCTTTGC |  |  |  |

Table S3 Supplementary material

| Sirex scaffold | E-value | Opsin | Organism | Accession number |
| --- | --- | --- | --- | --- |
| Scaffold_6 | 2.99E-160 | UVS | *A. mellifera* | NM_001011605.1 |
| Scaffold_6 | 1.91E-157 | UVS | *O. abietius* | KY368229.1 |
| Scaffold_7 | 2.24E-144 | LW1 | *A. mellifera* | NM_001011639.2 |
| Scaffold_7 | 1.29E-163 | LW1 | *O. abietius* | KY368227.1 |
| Scaffold_692 | 2.36E-137 | LW2 | *A. mellifera* | NM_001077825.1 |
| Scaffold_692 | 9.07E-141 | LW2 | *O. abietius* | KY368226.1 |
| Scaffold_502 | 7.56E-8 | SWS | *A. mellifera* | NM_001011606.1 |
| Scaffold_214 | 1.32E-12 | SWS | *O. abietius* | KY368228.1 |

Table S4 Supplementary material

| Order |  |  | Suborder | Infraorder | Superfamily | Family | Species | LW1 | LW2 | SW | UV |
| --- | --- | --- | --- | --- | --- | --- | --- | --- | --- | --- | --- |
| Hymenoptera | Eusymphyta |  |  |  | Tenthredinoidea | Diprionidae | *Neodiprion lecontei* | 1 | 1 | 1 | 1 |
| Hymenoptera | Eusymphyta |  |  |  | Tenthredinoidea | Tenthredinidae | *Athalia rosae* | 1 | 1 | 1 | 1 |
| Hymenoptera | Eusymphyta |  |  |  | Tenthredinoidea | Tenthredinidae | *Tenthredo koehleri* | 1 | 0 | 1 | 1 |
| Hymenoptera | Unicalcarida |  |  |  | Siricoidea | Siricidae | *Sirex noctilio* | 1 | 1 | 0 | 1 |
| Hymenoptera | Unicalcarida |  |  |  | Cephoidea | Cephidae | *Cephus cinctus* | 1 | 1 | 1 | 1 |
| Hymenoptera | Unicalcarida | Vespina |  |  | Orussoidea | Orussidae | *Orussus abietinus* | 1 | 1 | 1 | 1 |
| Hymenoptera | Unicalcarida | Vespina | Apocrita | Parasitoida | Chalcidoidea | Agaonidae | *Ceratosolen solmsi* | 1 | 1 | 1 | 1 |
| Hymenoptera | Unicalcarida | Vespina | Apocrita | Parasitoida | Chalcidoidea | Encyrtidae | *Copidosoma floridanum* | 1 | 1 | 1 | 1 |
| Hymenoptera | Unicalcarida | Vespina | Apocrita | Parasitoida | Chalcidoidea | Pteromalidae | *Nasonia vitripennis* | 1 | 1 | 1 | 1 |
| Hymenoptera | Unicalcarida | Vespina | Apocrita | Parasitoida | Chalcidoidea | Trichogrammatidae | *Trichogramma pretiosum* | 1 | 1 | 1 | 1 |
| Hymenoptera | Unicalcarida | Vespina | Apocrita | Parasitoida | Cynipoidea | Figitidae | *Leptopilina clavipes* | 1 | 1 | 1 | 1 |
| Hymenoptera | Unicalcarida | Vespina | Apocrita | Parasitoida | Ichneumonoidea | Braconidae | *Cotesia vestalis* | 1 | 1 | 1 | 1 |
| Hymenoptera | Unicalcarida | Vespina | Apocrita | Parasitoida | Ichneumonoidea | Braconidae | *Diachasma alloeum* | 1 | 1 | 1 | 1 |
| Hymenoptera | Unicalcarida | Vespina | Apocrita | Parasitoida | Ichneumonoidea | Braconidae | *Fopius arisanus* | 1 | 1 | 1 | 1 |
| Hymenoptera | Unicalcarida | Vespina | Apocrita | Parasitoida | Ichneumonoidea | Braconidae | *Microplitis demolitor* | 1 | 1 | 1 | 1 |
| Hymenoptera | Unicalcarida | Vespina | Apocrita | Aculeata | Chrysidoidea | Chrysididae | *Chrysis viridula* | 1 | 0 | 1 | 1 |
| Hymenoptera | Unicalcarida | Vespina | Apocrita | Aculeata | Vespoidea | Vespidae | *Poliste canadensis* | 1 | 1 | 1 | 1 |
| Hymenoptera | Unicalcarida | Vespina | Apocrita | Aculeata | Vespoidea | Vespidae | *Polistes dominula* | 1 | 1 | 1 | 1 |
| Hymenoptera | Unicalcarida | Vespina | Apocrita | Aculeata | Formicoidae | Formicidae | *Acromyrmex echinatior* | 1 | 1 | 1 | 1 |
| Hymenoptera | Unicalcarida | Vespina | Apocrita | Aculeata | Formicoidae | Formicidae | *Atta cephalotes* | 1 | 1 | 1 | 1 |
| Hymenoptera | Unicalcarida | Vespina | Apocrita | Aculeata | Formicoidae | Formicidae | *Camponotus atriceps* | 1 | 0 | 0 | 1 |
| Hymenoptera | Unicalcarida | Vespina | Apocrita | Aculeata | Formicoidae | Formicidae | *Camponotus floridanus* | 1 | 1 | 1 | 1 |
| Hymenoptera | Unicalcarida | Vespina | Apocrita | Aculeata | Formicoidae | Formicidae | *Camponotus rufipes* | 0 | 1 | 1 | 1 |
| Hymenoptera | Unicalcarida | Vespina | Apocrita | Aculeata | Formicoidae | Formicidae | *Cardiocondyla obscurior* | 1 | 0 | 1 | 1 |
| Hymenoptera | Unicalcarida | Vespina | Apocrita | Aculeata | Formicoidae | Formicidae | *Cataglyphis bombycinus* | 1 | 0 | 0 | 1 |
| Hymenoptera | Unicalcarida | Vespina | Apocrita | Aculeata | Formicoidae | Formicidae | *Cerapachys biroi* | 1 | 0 | 1 | 1 |
| Hymenoptera | Unicalcarida | Vespina | Apocrita | Aculeata | Formicoidae | Formicidae | *Harpegnathos saltator* | 1 | 1 | 1 | 1 |
| Hymenoptera | Unicalcarida | Vespina | Apocrita | Aculeata | Formicoidae | Formicidae | *Linepithema humile* | 1 | 1 | 1 | 1 |
| Hymenoptera | Unicalcarida | Vespina | Apocrita | Aculeata | Formicoidae | Formicidae | *Monomorium pharaonis* | 1 | 1 | 1 | 1 |
| Hymenoptera | Unicalcarida | Vespina | Apocrita | Aculeata | Formicoidae | Formicidae | *Myrmecia spp* | 28 |  |  |  |
| Hymenoptera | Unicalcarida | Vespina | Apocrita | Aculeata | Formicoidae | Formicidae | *Nothoyrmecia macrops* | 1 |  |  |  |
| Hymenoptera | Unicalcarida | Vespina | Apocrita | Aculeata | Formicoidae | Formicidae | *Pogonomyrmex barbatus* | 1 | 1 | 1 | 1 |
| Hymenoptera | Unicalcarida | Vespina | Apocrita | Aculeata | Formicoidae | Formicidae | *Solenopsis invicta* | 1 | 1 | 1 | 1 |
| Hymenoptera | Unicalcarida | Vespina | Apocrita | Aculeata | Formicoidae | Formicidae | *Vollenhovia emeryi* | 1 | 1 | 1 | 1 |
| Hymenoptera | Unicalcarida | Vespina | Apocrita | Aculeata | Formicoidae | Formicidae | *Wasmannia auropunctata* | 1 | 1 | 1 | 1 |
| Hymenoptera | Unicalcarida | Vespina | Apocrita | Aculeata | Apoidea | Apidae | *Apis cerana* | 1 | 1 | 1 | 1 |
| Hymenoptera | Unicalcarida | Vespina | Apocrita | Aculeata | Apoidea | Apidae | *Apis dorsata* | 1 | 1 | 1 | 1 |
| Hymenoptera | Unicalcarida | Vespina | Apocrita | Aculeata | Apoidea | Apidae | *Apis florea* | 1 | 1 | 1 | 1 |
| Hymenoptera | Unicalcarida | Vespina | Apocrita | Aculeata | Apoidea | Apidae | *Apis mellifera* | 1 | 1 | 1 | 1 |
| Hymenoptera | Unicalcarida | Vespina | Apocrita | Aculeata | Apoidea | Apidae | *Bombus impatiens* | 1 | 1 | 1 | 1 |
| Hymenoptera | Unicalcarida | Vespina | Apocrita | Aculeata | Apoidea | Apidae | *Bombus terrestris* | 1 | 1 | 1 | 1 |
| Hymenoptera | Unicalcarida | Vespina | Apocrita | Aculeata | Apoidea | Apidae | *Diadasia afflicta* | 1 | 1 | 0 | 0 |
| Hymenoptera | Unicalcarida | Vespina | Apocrita | Aculeata | Apoidea | Apidae | *Diadasia rinconis* | 1 | 1 | 0 | 0 |
| Hymenoptera | Unicalcarida | Vespina | Apocrita | Aculeata | Apoidea | Apidae | *Eufriesea mexicana* | 1 | 1 | 1 | 1 |
| Hymenoptera | Unicalcarida | Vespina | Apocrita | Aculeata | Apoidea | Apidae | *Euglossa dilemma* | 1 | 1 | 1 | 1 |
| Hymenoptera | Unicalcarida | Vespina | Apocrita | Aculeata | Apoidea | Apidae | *Habropoda laboriosa* | 1 | 1 | 1 | 1 |
| Hymenoptera | Unicalcarida | Vespina | Apocrita | Aculeata | Apoidea | Apidae | *Melipona quadrifasciata* | 1 | 1 | 1 | 1 |
| Hymenoptera | Unicalcarida | Vespina | Apocrita | Aculeata | Apoidea | Halictidae | *Dufourea novaeangliae* | 1 | 1 | 1 | 1 |
| Hymenoptera | Unicalcarida | Vespina | Apocrita | Aculeata | Apoidea | Halictidae | *Lasioglossum albipes* | 1 | 1 | 1 | 1 |
| Hymenoptera | Unicalcarida | Vespina | Apocrita | Aculeata | Apoidea | Megachilidae | *Megachile rotundata* | 1 | 1 | 1 | 1 |
| Hymenoptera | Unicalcarida | Vespina | Apocrita | Aculeata | Apoidea | Megachilidae | *Osmia rufa* | 1 | 1 | 0 | 0 |

Figure S1 Supplementary material

Figure S2 Supplementary material


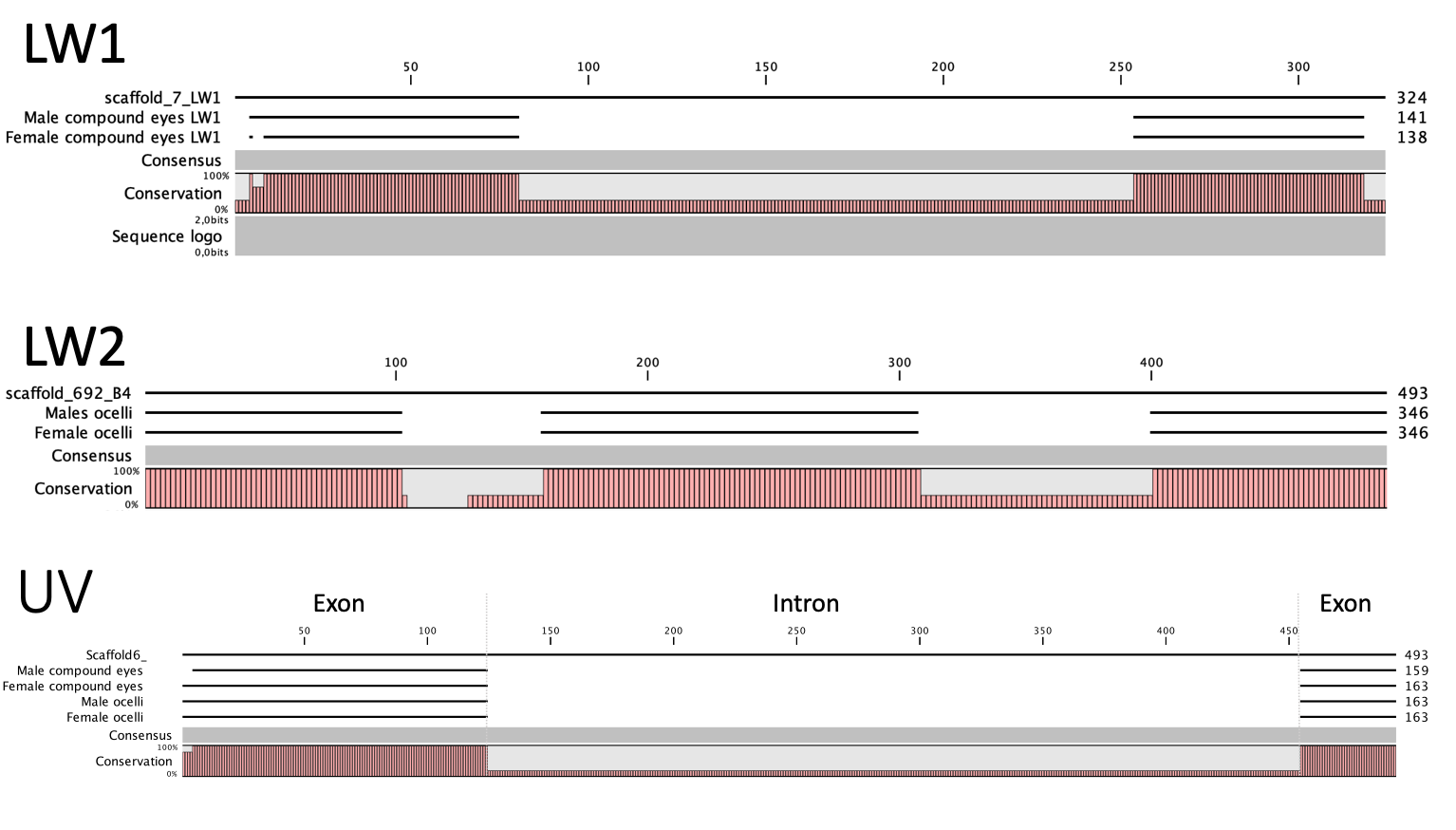

Supplement: Supplementary file 1 — Supplementary Information. [file 41598_2021_95107_MOESM1_ESM.docx]
